# Disentangling the lifestyle of bacterial communities in tropical soda lakes

**DOI:** 10.1038/s41598-022-12046-2

**Published:** 2022-05-13

**Authors:** Simone R. Cotta, Thierry A. Pellegrinetti, Ana Paula D. Andreote, Juliana S. Costa, Hugo Sarmento, Marli F. Fiore

**Affiliations:** 1 Center of Nuclear Energy in Agriculture (CENA/USP), University of São Paulo, Piracicaba, SP CEP: 13416-903 Brazil; 2 Department of Hydrobiology, Federal University of São Carlos, São Carlos, SP Brazil

**Keywords:** Water microbiology, Microbiome

## Abstract

Microbial lifestyles may reveal niche-specific signatures and can contribute to detecting the effects of abiotic fluctuations on biogeochemical cycles. Microorganisms make a tradeoff between optimizing nutrient uptake, improving biomass yield, and overcoming environmental changes according to environmental hostility. Soda lakes are natural environments rich in carbonate and bicarbonate water, resulting in elevated pH and salinities that frequently approach saturation. We hypothesized that during the dry period (elevated pH and salinity), microorganisms try to overcome this harshness by allocating energy to the cellular maintenance process. As these environmental conditions improve during the wet period, microorganisms will begin to invest in nutrient uptake. To test this hypothesis, we evaluated four soda lakes in two different seasons by applying metagenomics combined with flow cytometry (estimate heterotrophic bacterial biomass). The natural occurrence of cyanobacterial blooms in some lakes is the main driver of carbon. These primary producers provide organic carbon that supports heterotrophic bacterial growth and, consequently, a high biomass yield. Under harsh conditions (dry season), cyanobacteria invest in nutrient uptake mechanisms, whereas heterotrophic bacteria allocate energy to survive at the expense of biomass yield. Lakes without cyanobacteria blooms invest in nutrient uptake independent of environmental hostility. This study clarifies the microbial tradeoffs in hostile environments and the impact of this choice on carbon and energy flux in tropical alkaline lakes.

## Introduction

Knowledge of the microbial metabolic pathways may be critical for improving the prognosis of the effects of abiotic fluctuations on biogeochemical cycles. Under ever-changing environmental conditions in time and space, microorganisms must constantly adapt their functionality to guarantee continuity^1,2^.

Life strategies represent sets of traits that tend to correlate physiological or evolutionary tradeoffs with strategies that are favored under different environmental conditions^3,4^. Three main microbial life-history strategies were recently described by Malik and coworkers^4^; a high yield life strategy (Y), a resource acquisition life strategy (A), and a stress tolerance life strategy (S). The high-yield strategist maximizes resource uptake and allocates it to biosynthetic processes, whereas the resource acquisition strategist is based on competition for nutrient acquisition. Stress tolerance involves the production and secretion of components or cellular structures associated with overcoming stress conditions^4^.

The Brazilian Pantanal biome (specifically in the Nhecolândia sub-region) contains hundreds of soda lakes with high pH values (ranging from 9.5 to 11) and salinities approaching saturation. In contrast to other Pantanal sub-regions, Nhecolândia soda lakes are mainly influenced by the evaporation ratio (high water evaporation relative to precipitation, with no input from rivers or flooding)^5–7^. During the dry season, alkaline lakes remain isolated from the other lakes due to the low permeability of soil horizons (especially the silica layer), promoting solute concentration^8^. However, intensive rainfall (during the wet season) causes a rise in the water level on the subsurface that could connect these lakes, bypassing the silica layer^6,8^. Furthermore, these lakes are separated from the regional drainage system by “cordilheiras” (narrow and elongated sand hills covered by savanna vegetation) that are 2–3 m higher than adjacent plains. This barrier maintains alkaline waters and shows a high amount of organic matter that is more dependent on local cycles than terrestrial inputs by annual flooding^9,10^.

Despite harsh conditions (high pH and salinity), alkaline lakes are remarkably productive because of elevated temperatures and luminosity^11^. These lakes host diverse microbial communities and frequently experience seasonal or permanent cyanobacterial blooms^12,13^. These cyanobacterial blooms generate a pulse of organic carbon that affects the carbon turnover^14^. Approximately 20% of photosynthetic carbon is released in aquatic systems, and this organic matter supports a substantial portion of the heterotrophic community^15–17^. Photosynthetic primary production is the driving force behind nutrient recycling in marine and freshwater environments^16,18^. However, the carbon and energy fluxes in alkaline lakes have not been explored. In a hostile environment, it has been suggested that bacterial growth efficiency (BGE) is reduced, and energy is allocated to non-growth reactions that help cells maintain their molecular, cellular, and functional integrity, an equivalent of the S-strategy^4,18^.

Based on this, we hypothesized that microorganisms try to overcome this harshness during the dry season to allocate energy to the cellular maintenance process. The improvement in these environmental conditions during the wet season allows the microorganism to invest in nutrient uptake. To test this hypothesis, we evaluated four shallow, alkaline lakes on the Pantanal during two seasons, wet and dry, by applying metagenomics and flow cytometry.

## Results

Here, we adopted metagenomic and flow cytometry approaches to estimate the lifestyle of bacterial populations that allow them to overcome the harsh conditions observed in tropical soda lakes.

### Metagenomic datasets

After trimming and removing low-quality sequences, 39,324,182 to 48,803,399 million reads were recovered during the dry season, and 34,694,196 to 41,212,766 million reads were recovered during the wet season. The replicates were reproducible, showing slight variation in the number of reads obtained (data not shown).

### Bacterial communities’ composition in Nhecolândia alkaline shallow lakes

**Figure 1:**
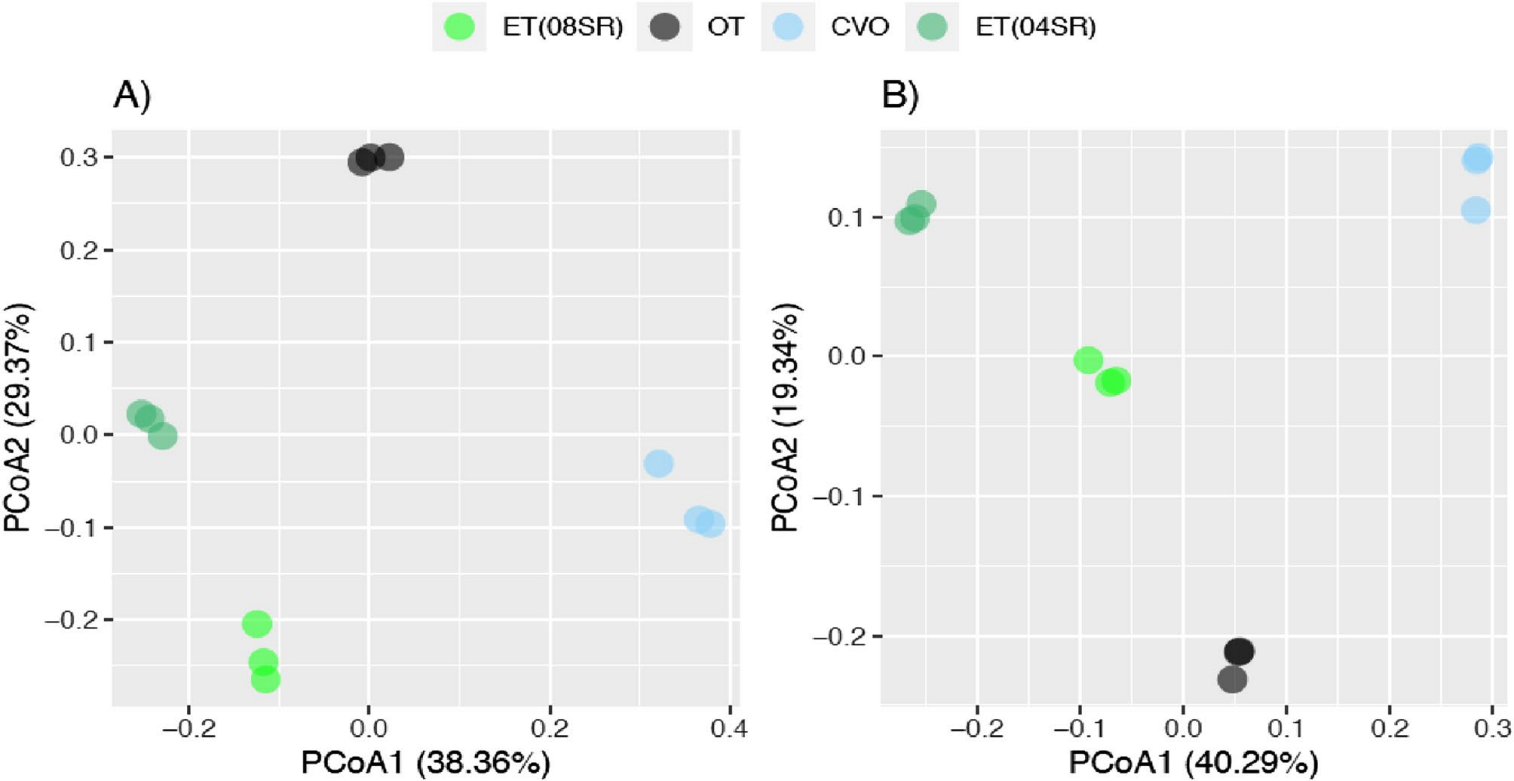
The Principal Coordinates Analysis (PCoA) of shallow alkaline lakes. The letter (**A**) corresponds to the dry season, while the letter (**B**) corresponds to the wet season.

The bacterial communities in the alkaline lakes were structurally different (Fig. 1). During the dry season (Fig. 1A), the first axis separated the lakes with the occurrence of cyanobacterial blooms from the lakes without the bloom (38.36%). The second axis separated lake OT (oligotrophic turbid) from the other lakes by 29.37% (ANOSIM, global R = 1.00; *p* value: 1e−04, PERMANOVA, R^2^ = 0.79881; *p* = 0.0001) (Fig. 1A). This pattern was similar during the wet season (Fig. 1B). The first axis separated the lakes without bloom (OT and CVO, clear vegetated oligotrophic lake) from the lakes with the occurrence of blooms [ET—eutrophic turbid lake (04SR) and ET (08SR)] by 40.28%. The second axis separated lake CVO from the other lakes by 19.34% (ANOSIM, global R = 1.00; *p* value: 2e−04; PERMANOVA, R^2^ = 0.75129; *p* value = 0.0001) (Fig. 1B).

At a high phylogenetic level, the lakes harbor a similar bacterial community composition. Actinobacteria, Bacteroidetes, Chloroflexi, Cyanobacteria, Firmicutes, Planctomycetes, Proteobacteria, Tenericutes, and Verrucomicrobia were the most abundant phyla identified in the alkaline lakes (relative abundance > 2%). The bacterial composition was similar between the lakes and seasons (Supplementary Figure 1). The differences observed in the PCoA analysis described above could be explained by the differential abundance of some bacterial groups.

### Differential abundance of bacterial community

**Figure 2:**
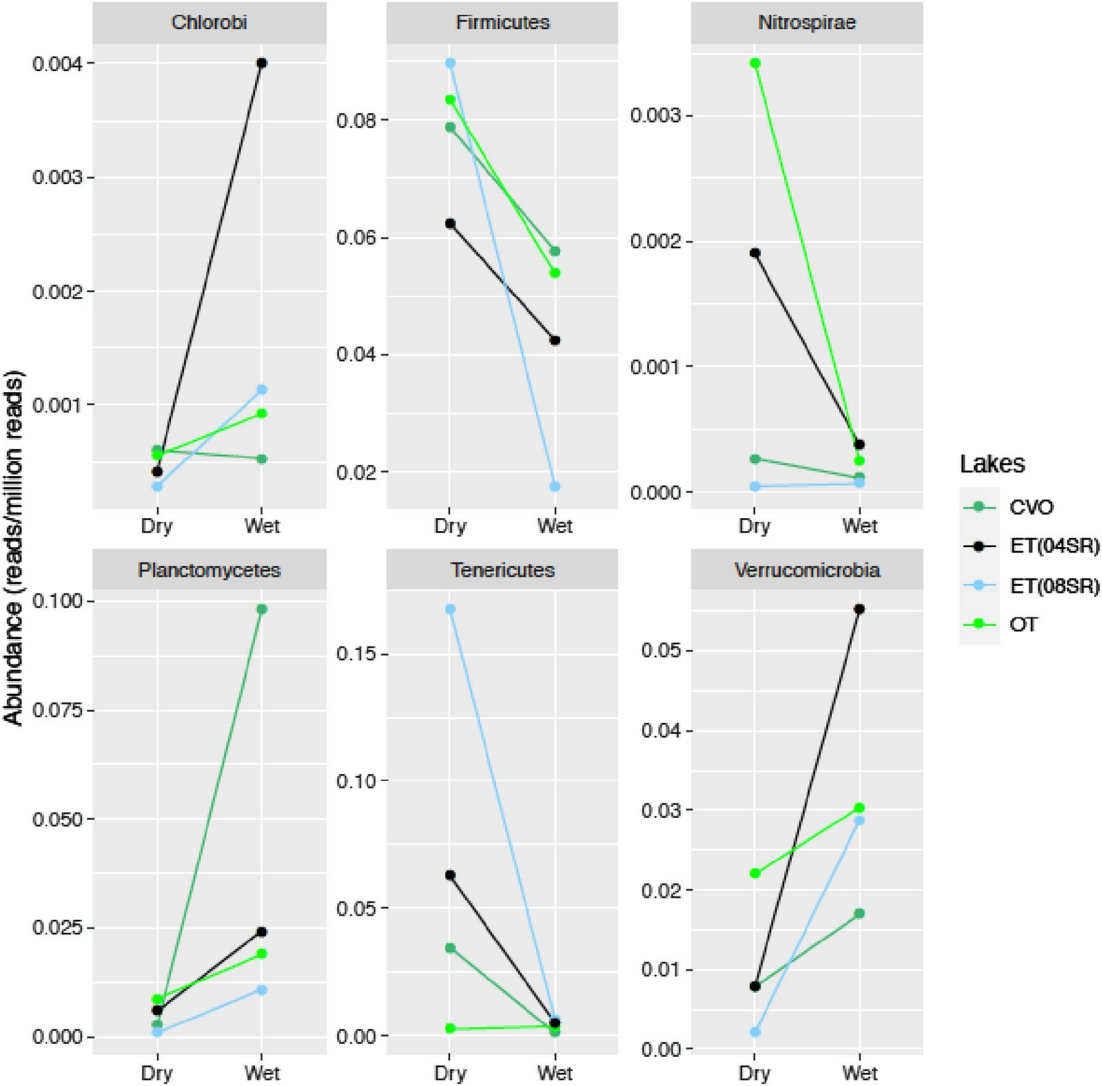
The differential abundances of bacterial groups associated with seasonality per lake. It was represented only in the groups whose abundance differed significantly between the harvest period (*p* < 0.05).

Although the bacterial composition was similar between lakes and between the seasons evaluated, it was possible to detect some groups whose abundance varied between the lakes and over time. Considering the bacterial differential abundance detected between the seasons, it is interesting that some groups increased during the dry season while others were enriched during the wet season. Chlorobi, Planctomycetes, and Verrucomicrobia were enriched during the wet period, whereas Firmicutes, Nitrospirae, and Tenericutes were more abundant during the dry season (Fig. 2).

During the dry season, the abundances of Actinobacteria, Bacteroidetes, Cyanobacteria, Fusobacteria, Lentisphaerae, Nitrospira, Tenericutes, and Verrucomicrobia varied among the lakes. Actinobacteria, Bacteroidetes, Fusobacteria, Lenthisphaerae, and Tenericutes were abundant in lakes with blooms (ET), while Actinobacteria, Nitrospirae, and Verrucomicrobia were abundant in lakes without blooms (CVO and OT). The abundance of Cyanobacteria was reduced in lake CVO (Supplementary Figure 2).

During the wet season, the abundance of Acidobacteria, Actinobacteria, Chloroflexi, Cyanobacteria, Firmicutes, Gemmatimonadetes, and Proteobacteria fluctuated between lakes. Acidobacteria, Actinobacteria, Chloroflexi, Gemmatimonadetes, and Proteobacteria were more abundant in lakes without blooms, while Cyanobacteria were abundant in lakes with blooms. Firmicutes were reduced in the ET (08SR) lake (Supplementary Figure 3).

### Correlation between abiotic and biotic factors

Chemical and physical characteristics were relevant factors that explained the changes in bacterial community composition and functionality. Here, only the environmental variables significantly explained the variability in bacterial community structure based on db-RDA analysis (data not shown). For the dry season, the environmental variables that best explained the variability in the bacterial community were chlorophyll-*a*, TP, TPN, Si, and Ni (*p* < 0.05), while for the wet season, the environmental variables were E.C., pH, Fe, Ni, and NO_2_^−^ (*p* < 0.05). These variables were correlated with the differentially abundant bacterial groups previously described.

**Figure 3:**
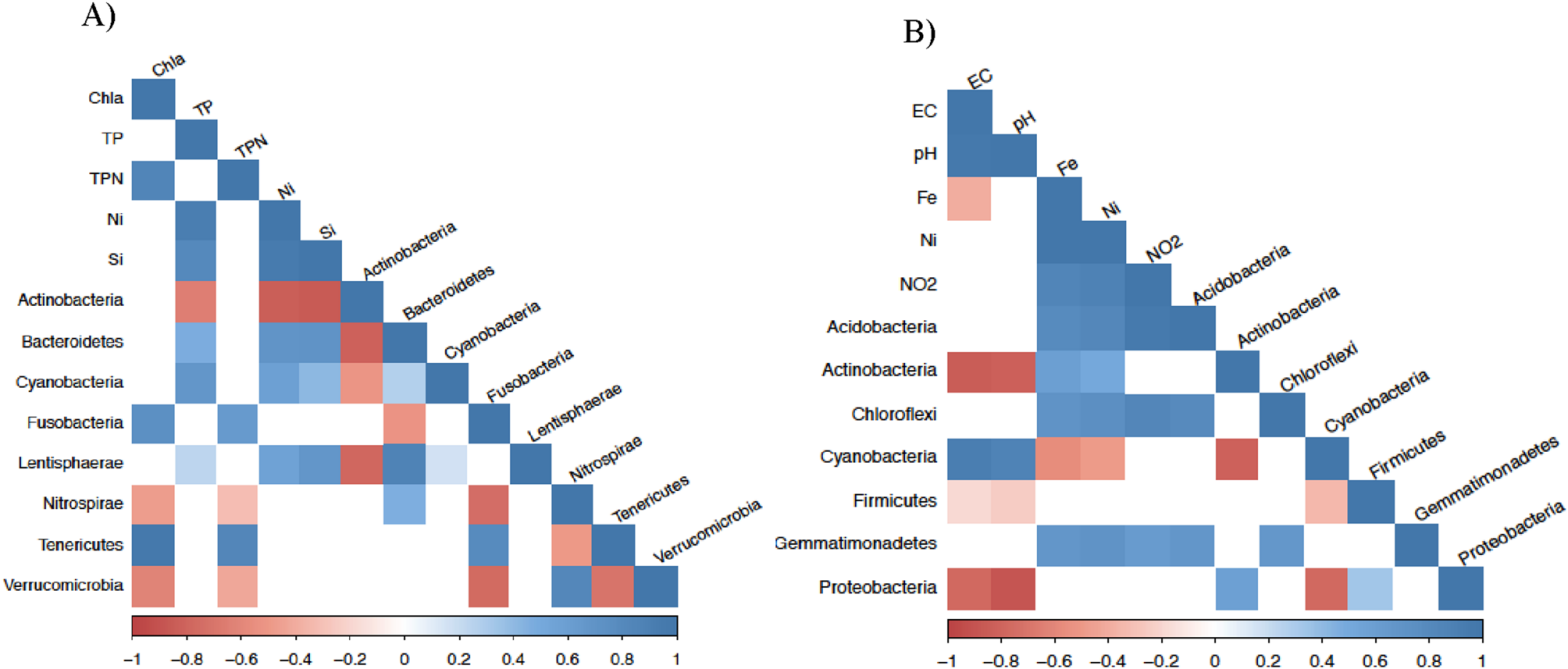
The correlation plot between abiotic parameters with differential abundant groups. The blue squares represent positive correlations, and the red squares represent the negative correlations. The white squares represent the absence of significant correlation (*p* < 0.05). The letter (**A**) corresponds to the dry period, while the letter (**B**) corresponds to the wet period.

During the dry season, Actinobacteria, Nitrospirae, and Verrucomicrobia were negatively correlated with the evaluated environmental features. Actinobacteria were negatively correlated with TP, Ni, and Si, whereas Nitrospirae and Verrucomicrobia were negatively correlated with chlorophyll-a and TPN (Fig. 3A). Bacteriodetes, Cyanobacteria, Fusobacteria, Lenthisphaerae, and Tenericutes were positively correlated with the chemical characteristics of the lakes. Bacteriodetes, Cyanobacteria, and Lenthisphaerae were positively correlated with TP, Ni, and Si. Fusobacteria and Tenericures were positively correlated with chlorophyll-a and TPN (Fig. 3A).

As observed in the dry season, in the wet season, some bacterial groups were positively correlated with some abiotic conditions, while others were negatively correlated. Acidobacteria, Chloroflexi, and Gemmatimonadetes were positively correlated with Fe, Ni, and NO_2_^−^. Actinobacteria were positively correlated with Fe and Ni but negatively correlated with E.C. and pH; an opposite trend was observed for Cyanobacteria. The phylum Cyanobacteria was positively correlated with E.C. and pH, and negatively correlated with Fe and Ni. Firmicutes and Proteobacteria were negatively correlated with E.C. and pH (Fig. 3B).

### Distribution of prevalent functions on alkaline lakes

The prevalent functions (> 3%) associated with alkaline lakes were Carbohydrates, Protein Metabolism, and Amino Acids (and their derivatives) (Supplementary Figure 4). Following these functions, RNA Metabolism, Respiration, Nucleosides and Nucleotides, Cell Wall and Capsule, cofactors (cofactors, vitamins, prosthetic group, pigments), DNA metabolism, and Membrane transport were also abundant. The Phages, Prophages, Transposable elements, and Plasmids categories were prevalent specifically for lake ET(08SR) during the dry season (Supplementary Figure 4).

### Bacterial trait-based framework

**Figure 4:**
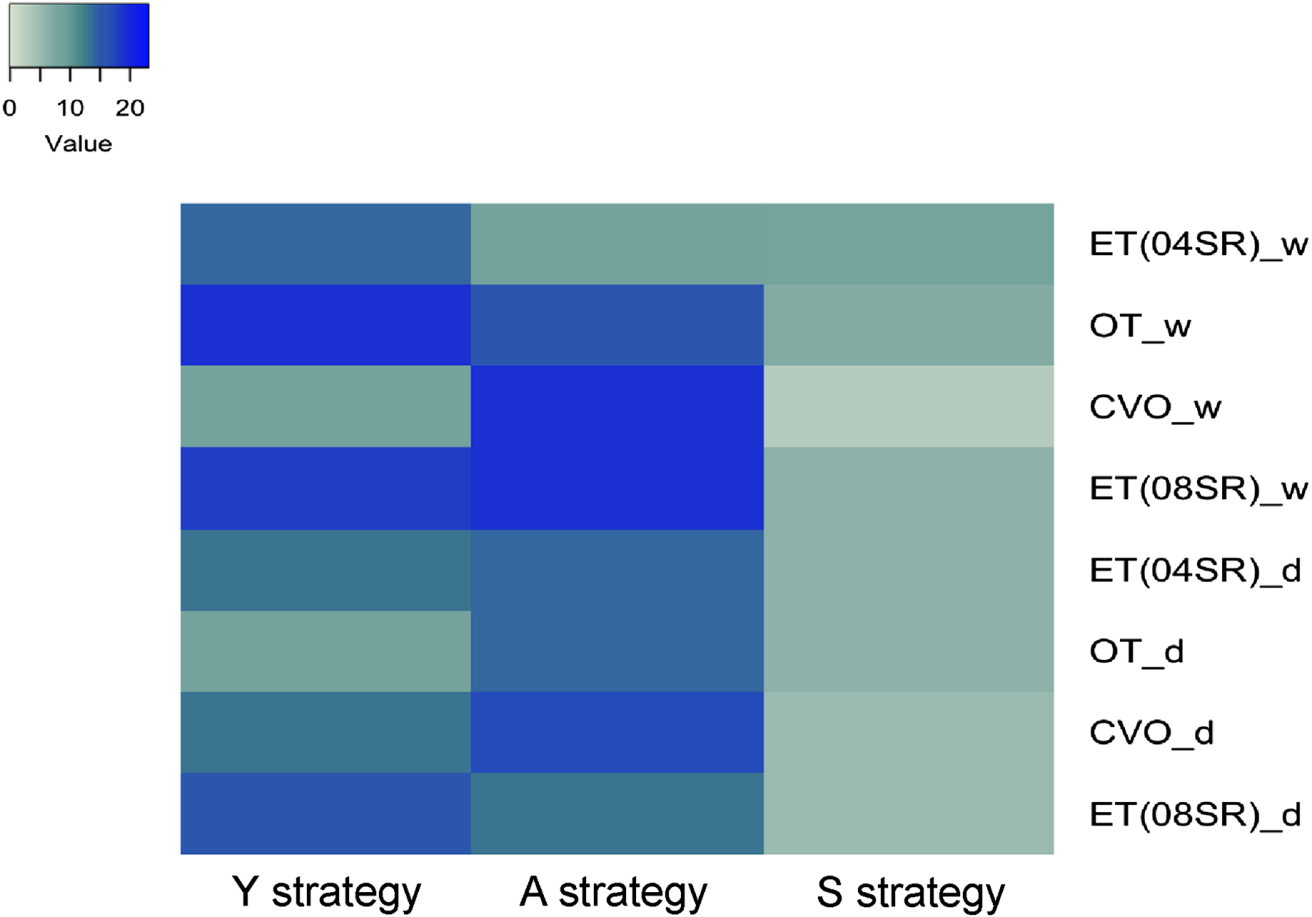
Heatmap of the number of traits affiliated with each life strategy. The letters D and W represent samples from the dry and wet seasons, respectively.

Based on the trait-based framework (the statistically significant functions were grouped following the categorization suggested in Supplementary Table 01), it was possible to observe that seasonality and the presence of bloom had a remarkable effect on the bacterial community tradeoff. Considering the whole bacterial community, inhabiting organisms of lakes OT and CVO preferentially adopted an A strategy (e.g., enrichment of ABC transporters), while the local bacterial community on ET lake preferentially adopted a Y strategy (e.g., enrichment of Di—oligosaccharides function) during the dry season (Fig. 4; Supplementary Table 02). However, this tradeoff was modified in the wet season, when the bacterial community on the lakes preferentially adopts a Y strategy (e.g., enrichment of aminosugar function). The exception was the lake CVO, where the dwelling bacterial community continued to adopt an A strategy (e.g., enrichment of protein translocation in the plasmatic membrane and sugar phosphotransferase functions) (Supplementary Table 02; Fig. 4).

To understand how bacterial communities are affected by the presence of Cyanobacteria, we removed sequences associated with this phylum to perform a trait-based framework analysis. After removing the Cyanobacteria sequences from the analysis, it is interesting to note that traits associated with the S strategy were enriched in ET (04SR) and OT lakes during the dry season. In the wet season, the bacterial community tradeoff was similar to that observed in the presence of Cyanobacteria (Supplementary Figure 5A and C). The preferential use of Y-strategies in ET(04SR) and 08SR lakes was corroborated by the results obtained by determining heterotrophic bacterial biomass. Lakes where blooms occurred [ET(04SR) and ET(08SR)] had a higher biomass of heterotrophic bacteria than the other lakes, and this was independent of seasonality (Supplementary Figure 5B and D). Furthermore, absolute quantification of the microbial community by flow cytometry showed that during the dry period, heterotrophic bacteria (HB) were abundant in ET lakes compared to OT and CVO lakes. This pattern remained in the wet period when HB was prevalent, while OT Lake showed the lowest abundance. Notably, picoeukaryotes were prevalent in the CVO Lake during the wet period (Table 1).

**Table 1:** Main limnological parameters of shallow alkaline lakes.

	ET(04SR)_D	OT_D	CVO_D	ET(08SR)_D	ET(04SR)_W	OT_W	CVO_W	ET(08SR)_W
pH	10.01 ± 0.01^b^	9.69 ± 0.03^c^	8.55 ± 0.05^d^	10.07 ± 0.05^a^	10.0 ± 0.05^a^	9.08 ± 0.07^c^	8.63 ± 0.05^d^	9.41 ± 0.05^b^
Temperature (°C)	26.43 ± 0.11^Ac^	37.66 ± 0.16^Ab^	27.25 ± 0.01^Ad^	35.05 ± 0.13^Aa^	25.74 ± 0.90^Ba^	26.25 ± 0.45^Bc^	27.19 ± 0.21^Bd^	25.41 ± 0.21^Bb^
E.C. (μS cm−1)	16,295 ± 557^Ac^	17,990 ± 65^Ab^	1777 ± 0^Ad^	39,257 ± 194^Aa^	1723 ± 4^Ba^	679.0 ± 7^Bc^	556.6 ± 0.57^Bd^	1001 ± 13^Bb^
Salinity (g L^−1^)	11.10 ± 1.74^Ab^	9.71 ± 0.62^Ab^	0.98 ± 0.15^Ac^	25.12 ± 2.14^Aa^	1.69 ± 0.13^Ba^	0.82 ± 0.003^Bb^	0.53 ± 0.005^Bc^	0.96 ± 0.02^Bb^
Alkalinity (mmol L^−1^)	97.5 ± 3.81^Ab^	66.95 ± 2.72^Ac^	8.03 ± 0.35^Ad^	200.02 ± 8.85^Aa^	8.77 ± 1.59^Ba^	3.80 ± 0.28^Bbc^	3.80 ± 0.28^Bc^	5.13 ± 0.28^Bb^
NH_4_^+^ (mg L^−1^)	4.68 ± 1.49^Aa^	0.29 ± 0.17^Ab^	0.31 ± 0.14^Ab^	2.75 ± 0.24^Aa^	0.77 ± 0.007^Ba^	0.03 ± 0.001^Bbc^	0.04 ± 0.01^Bc^	0.07 ± 0.01^Bb^
NO_2_^−^ (mg L^−1^)	0.17 ± 0.04^Ac^	0.33 ± 0.02^Ab^	0.01 ± 0.001^Ad^	0.66 ± 0.04^Aa^	0.03 ± 0.02^Bb^	0.08 ± 0.0009^Ba^	0.003 ± 0^Bc^	0.003 ± 0^Bc^
NO_3_^−^ (mg L^−1^)	0.78 ± 0.007^Ac^	0.84 ± 0.08^Ab^	0.05 ± 0.01^Ad^	0.22 ± 0.06^Aa^	0.12 ± 0.01^Bb^	0.25 ± 0.07^Ba^	0.006 ± 0^Bc^	0.03 ± 0.04^Bc^
Cl^−^ (mg L^−1^)	381.76 ± 94^Ab^	529.43 ± 105^Ab^	39.16 ± 3.87^Ac^	1056.5 ± 134^Aa^	41.77 ± 8^Ba^	20.05 ± 1^Bbc^	6.88 ± 0.37^Bc^	28.04 ± 13^Bab^
oPO_4_^–3^ (mg L^−1^)	84.09 ± 4.05^Aa^	86.91 ± 11^Aa^	0.41 ± 0.05^Ac^	60.42 ± 3.72^Ab^	2.2 ± 0.17^Ba^	1.16 ± 0.32^Bb^	0.07 ± 0.04^Bc^	0.43 ± 0.05^Bc^
Na^+^ (mg L^−1^)	6,735 ± 768^Ab^	6,018 ± 1125^Ab^	432.72 ± 27^Ab^	14,370 ± 893^Aa^	581.45 ± 158^Ba^	215.8 ± 5.88^Bbc^	81.95 ± 13^Bc^	226.35 ± 22^Bb^
SO_4_^–2^ (mg L^−1^)	20.93 ± 2.48^Ac^	809.3 ± 190^Ab^	2.79 ± 1.65^Ad^	1,339 ± 172^Aa^	0.01 ± 0^Bc^	21.91 ± 3^Bc^	0.63 ± 1^Bc^	13.20 ± 8^Bb^
Ca^+2^ (mg L^−1^)	316.4 ± 76^Bb^	581 ± 311^Bb^	81.87 ± 9^Bb^	407.1 ± 211^Ba^	69.5 ± 5.91^Aa^	79.72 ± 48^Aa^	40.28 ± 7^Ab^	55.33 ± 10^Aab^
DOC (mg L^−1^)	0.92 ± 0.17^Ab^	0.30 ± 0.02^Ac^	0.07 ± 0.005^Ad^	5.32 ± 0.18^Aa^	77.53 ± 0.12^Ba^	12.12 ± 0.40^Bd^	15.94 ± 1.37^Bc^	39.94 ± 0.39^Bb^
DIC (mg L^−1^)	1.20 ± 0.02^Ab^	1.07 ± 0.02^Ac^	0.17 ± 0.002^Ad^	1.50 ± 0.04^Aa^	171.59 ± 1.68^Ba^	54.46 ± 0.58^Bc^	47.87 ± 1.78^Bd^	91.75 ± 0.44^Bb^
TDN (mg L^−1^)	1389 ± 68^Ab^	177.17 ± 31^Ac^	358.6 ± 11^Ac^	2819 ± 265^Aa^	10.52 ± 0.54^Ba^	0.98 ± 0.02^Bc^	1.25 ± 0.07^Bc^	4.24 ± 0.05^Bb^
TP (mg L^−1^)	179.1 ± 0.65^Ab^	155.2 ± 11^Ac^	9.43 ± 0.57^Ad^	207.3 ± 1.13^Aa^	3.4 ± 0.34^Ba^	1.16 ± 0.32^Bb^	0.07 ± 0.04^Bc^	0.43 ± 0.05^Bc^
TN (mg L^−1^)	1,511 ± 14^Ab^	291.47 ± 19^Ac^	419.9 ± 35^Ac^	3,394 ± 131^Aa^	16.12 ± 2.63^Ba^	1.95 ± 0.10^Bc^	2.23 ± 0.09^Bbc^	5.62 ± 0.47^Bb^
Chlorophyll-a (μg L^−1^)	2046 ± 958^Ab^	0.01 ± 0^Ab^	0.22 ± 0.10^Ab^	11,348 ± 1.47^Aa^	119.3 ± 3.31^Ba^	9.76 ± 0.31^Bc^	6.07 ± 1.05^Bc^	37.68 ± 3^Bb^
TPN (mg L^−1^)	121.38 ± 72^A^	114.29 ± 27^A^	61.26 ± 25^A^	574.7 ± 365^A^	5.62 ± 2.54^Ba^	0.97 ± 0.09^Bb^	0.97 ± 0.09^Bb^	1.38 ± 0.52^Bb^
DIN (mg L^−1^)	5.63 ± 1.53^Ab^	1.47 ± 0.23^Ab^	0.38 ± 0.13^Ac^	3.64 ± 0.29^Aa^	0.94 ± 0.02^Ba^	0.38 ± 0.07^Bb^	0.04 ± 0.01^Bc^	0.10 ± 0.05^Bc^
DON (mg L^−1^)	1384 ± 70^Ab^	175.7 ± 32^Ad^	358.3 ± 11^Ac^	2,815 ± 265^Aa^	9.55 ± 0.56^Ba^	0.60 ± 0.08^Bc^	1.20 ± 0.09^Bc^	4.13 ± 0.11^Bb^
Bacterioplankton (cell mL^−1^)	5.86 × 10^8^ ± 2.16	1.65 × 10^7^ ± 5.26	5.35 × 10^6^ ± 3.55	1.90 × 10^8^ ± 8.47	9.03 × 10^7^ ± 2.92	6.97 × 10^3^ ± 3.22	2.33 × 10^6^ ± 6.75	1.21 × 10^7^ ± 1.73
**Phytoplankton categories (cell mL**^**−1**^**)**								
PcyPc	1.58 × 10^5^ ± 2.20	2.66 × 10^6^ ± 2.12	1.30 × 10^4^ ± 5.72	4.42 × 10^5^ ± 3.32	9.10 × 10^5^ ± 1.19	1.31 × 10^3^ ± 9.72	3.97 × 10^3^ ± 6.10	1.02 × 10^5^ ± 6.59
Peuk	1.16 × 10^4^ ± 1.23	2.71 × 10^4^ ± 2.38	1.65 × 10^3^ ± 1.13	5.37 × 10^5^ ± 4.36	8.25 × 10^3^ ± 4.91	2.93 × 10^3^ ± 8.90	2.84 × 10^3^ ± 1.98	1.34 × 10^4^ ± 7.81
PE rich euk or cyano	1.37 × 10^6^ ± 2.59	5.93 × 10^3^ ± 8.72	7.28 × 10^2^ ± 5.44	1.45 × 10^6^ ± 4.50	1.69 × 10^6^ ± 5.18	9.35 × 10^1^ ± 3.75	9.72 × 10^1^ ± 1.35	6.62 × 10^4^ ± 2.82
PcyPE_2	1.91 × 10^6^ ± 5.35	1.93 × 10^3^ ± 7.06	5.69 × 10^3^ ± 6.48	1.23 × 10^3^ ± 4.27	3.67 × 10^1^ ± 4.23	5.50 ± 7.02	1.28 × 10^3^ ± 2.20	5.50 ± 7.02
PcyPE_1	0	3.20 × 10^5^ ± 8.96	6.39 × 10^3^ ± 2.39	2.31 × 10^5^ ± 1.29	1.38 × 10^6^ ± 2.98	1.49 × 10^3^ ± 7.91	2.03 × 10^3^ ± 4.71	2.22 × 10^5^ ± 8.84
Neuk	6.42 × 10^2^ ± 2.60	7.41 × 10^2^ ± 1.95	8.30 × 10^2^ ± 1.28	2.47 × 10^2^ ± 4.27	5.50 × 10^1^ ± 1.10	2.99 × 10^2^ ± 3.15	1.06 × 10^2^ ± 4.88	9.58 × 10^4^ ± 1.02

The letter D correspond to the dry season while the letter W correspond to the wet season. The uppercase letters compare the seasons (dry and wet), while lowercase letters compare the lakes (*p* < 0.05).

*E.C* electric conductivity, *DOC* dissolved organic carbon, *DIC* dissolved inorganic carbon, *TDN* total dissolved nitrogen, *TP* total phosphorus, *TN* total nitrogen, *TPN* total particulate nitrogen, *DIN* dissolved inorganic nitrogen, *DON* dissolved organic nitrogen, *PcyPC* phycocyanin-rich picocyanobacterial, *Peuk* picoeukaryote, *PE rich euk or cyano* phycoerythrin rich eukaryote or cyanobacteria, *PcyPE_1 and PcyPE_2* phycoerythrin-rich picocyanobacterial type I and II, *Neuk* nanoeukaryote.

## Discussion

The associated microbiome of alkaline lakes has been described in several ecosystems worldwide. In general, the major phyla associated with alkaline lakes were Actinobacteria, Bacteriodetes, Chloroflexi, Cyanobacteria, Firmicutes, Planctomycetes, Proteobacteria, Spirochaetes, Tenericutes, and Verrucomicrobia, a similar bacterial community composition observed on this study^12,19^. This similar frequency of bacterial community composition could be associated with niche conservatism, whereby species present traits that allow them to cope with certain environmental conditions^20^.

However, this bacterial composition pattern was not static. It is possible to detect fluctuations in bacterial abundance and composition over time and space. This shift is associated with the selection of populations that are more suitable for the given abiotic conditions^1^. During the dry season, it was possible to observe an increase in nutrient and ion concentrations owing to the water evaporation rate. This increase in nutrient concentrations allows organisms to grow in environments where resources are abundant^21^. Usually, these organisms have high growth and metabolic rates, which can be disadvantageous in stable, nutrient-poor environments^21^.

During the dry season, a differential abundance of Firmicutes, Nitrospirae, and Tenericutes was observed. Specifically, Nitrospirae was abundant in Lake OT, where water showed a high concentration of particulate material resulting in a black color (low irradiance). During this season, this lake had a high concentration of nitrite and nitrate, which are important elements associated with the physiology of this bacterial group^13^. Organisms belonging to the phylum Tenericutes are frequently described as obligate symbionts because of their small genomes. However, they are resistant to osmotic lysis and show an enrichment in DNA repair mechanisms on their genomes, a feature associated with stress tolerance^22^. Interestingly, Tenericutes was positively correlated with chlorophyll-a, indicating a possible association with phytoplankton (mainly cyanobacteria).

However, the increase in rainfall during the wet season results in the dilution of nutrients in alkaline lakes, promoting a shift in bacterial abundance and composition. The reduction in nutritional status selects for organisms well-adapted to nutrient-poor environments, where resource uptake is prioritized in relation to biomass growth^21^. Chlorobi, Planctomycetes, and Verrucomicrobia were enriched during this period. Although the nutritional status was reduced during the wet season, the pH increased slightly. This increase in pH promotes an increase in organic carbon availability, which could stimulate Planctomycetes metabolism^23,24^. Planctomycetes have slow growth taxa that compensate for this through the efficient use of organic matter^24^.

This increase in pH could be associated with the carbon concentration mechanism (CCM). Some autotrophic organisms, especially Cyanobacteria, enhance carbon fixation during photosynthesis through the uptake of inorganic carbon (CO_2_, HCO^−^_3_, and CO_2_^–3^)^25,26^. As a result, the environmental pH increases owing to the excretion of OH- and the generation of pericellular CaCO_3_ precipitation^27,28^. Cyanobacteria are described as environmental engineers because they have strong effects on higher trophic levels and ecosystems functioning as critical drivers of bacterial assembly.

Microorganisms exhibit versatile metabolism, and this variability modulates the organization and functioning of communities. The trait-based approach, which analyses trait variation, is widely being adopted in ecology because it can clarify the microbial adaptations that permit the colonization of a specific niche and how these organisms will respond to environmental change^29,30^. Some criteria have been suggested to organize and classify the traits in the function of different microbial lifestyle strategies. However, this is not a consensus, and it is continuously updating^4,31^.

Resource utilization and competition for nutrients are important factors that shape phytoplankton communities^32,33^. Resource availability in the aquatic environment is directly associated with spatiotemporal variations and is dependent on the quantity and quality of these resources. During the dry season, the bacterial community preferentially adopted an A-strategy [except for ET(08SR) lake], wherein the bacterial groups enhance nutrient acquisition at the expense of growth yield^4^. Although a high nutrient concentration was promoted by the high evaporation ratio, the quality and availability of these nutrients could be low, enhancing the necessity to efficiently uptake nutrients rather than microbial biomass production.

Notably, the tradeoff between nutrient uptake and biomass production was modified if the target was exclusively the heterotrophic bacterial community. Heterotrophic bacteria preferentially adopt an S-strategy when subjected to hostile environments. This tradeoff results in a direct energy flux for cell maintenance at the expense of bacterial growth efficiency (BGE). This mechanism is well known in freshwater and marine environments^18^. Therefore, the adoption of the A-strategy by the whole bacterial community during the dry season is predominantly associated with cyanobacteria. The CCM mechanism described above is an important process of CO_2_ uptake and an important strategy for adapting to the major changes in CO_2_ availability that can be encountered during cyanobacteria blooms^34^.

However, these microbial tradeoffs change during the wet season, and this change represents niche differentiation among species, which emerges from individual-level constraints within an environmental context^35^. During the wet season, microbial communities adopt the Y-strategy, especially those inhabiting lakes with blooms. Primary productivity drives the energy flux through food webs, supporting the respiration and yield of heterotrophic bacteria^36^. Lakes with cyanobacterial blooms showed eutrophic characteristics, such as a high concentration of carbon, nitrogen, and phosphorus nutrients, which stimulate the microbial community to grow and consequently increase biomass production^37^.

## Conclusion

Therefore, cyanobacterial blooms mediate carbon and flux energy in tropical alkaline lakes. During the dry season, cyanobacteria can adapt to harsh environmental conditions (e.g., high UV irradiation) through CO_2_ uptake mediated by the CCM mechanism. As a consequence, these alternative processes for CO_2_ fixation could promote alkalization of the water, driving heterotrophic bacteria to adopt a strategy focused on maintaining cellular functioning over the biomass yield. When environmental conditions become more favorable during the wet period, cyanobacteria support bacterial growth. This “cyanobacteria factor” is evident in the CVO lake where cyanobacteria are absent. Independent of the sampling period, the heterotrophic bacterial community inhabiting the CVO lake took up nutrients to support cellular functioning, which compromised biomass yields.

## Material and methods

### Data collection

We evaluated four lakes located in the São Roque Reserve in the Nhecolândia sub-region of Mato Grosso do Sul State, Brazil. Recently, soda lakes found in this region were categorized into three groups: eutrophic turbid (ET), oligotrophic turbid (OT), and clear vegetated oligotrophic (CVO)^38^. ET lakes presented a natural cyanobacterial bloom from *Anabaenopsis elenkinii* (Nostocales) or *Arthrospira platensis* (Oscillatoriales) species, resulting in greenish waters. We selected two lakes belonging to the ET group (04SR and 08SR), one belonging to the OT group (06SR), and one belonging to the CVO group (07SR). Samples were collected from each lake with replicates separated by at least 50 m during the dry period and approximately 100 m during the wet period.

The physical and chemical features of water were defined as described by Pellegrinetti et al.^38^. Briefly, total nitrogen (TN) and total phosphorus (TP) contents were simultaneously detected using the persulfate method. Dissolved organic and inorganic carbon (DOC and DIC, respectively) and total dissolved nitrogen (TDN) were quantified by combustion (Shimadzu model TOC-5000A analyzer). The ion concentrations (NH_4_^+^, NO_3_^−^, and NO_2_^−^) were determined by flow injection analyses. Orthophosphate (oPO_4_^3−^) concentrations were quantified using the ascorbic acid method. Alkalinity was analyzed using 0.1 mol L^−1^ hydrochloric acid solution titration. Total dissolved solids were determined using the Environmental Protection Agency method 1684. Water salinity was estimated from the total amount of dissolved inorganic solids in the water samples. Concentrations of Na^+^, K^+^, Mg^2+^, Ca^2+^, Cl^−^, and SO_4_^2−^ were analyzed using ICS-90 ion chromatography. Trace elements, such as Al, B, Cu, Fe, Mn, Ni, Si, and Zn were determined by inductively coupled plasma optical emission spectrometry (ICP-OES; JY ULTIMA 2000, Longjumeau, France)^38^. The main physical and chemical characteristics of the samples are listed in Table 1.

As seasonality is an important phenomenon, sampling was conducted in October 2017 during the dry season and in September 2018 during the wet season. The seasonal rainfall cycle frequently concentrates on the rain between September and April (http://www.dsr.inpe.br/laf/series/). During the dry season, these lakes presented low depth (0.01–0.05 m), an apparent absence of stratification, widths ranging from 100 to 220 m, and lengths ranging from 100 to 450 m. In the wet season, these lakes also present an absence of stratification but an increase in depth (0.08–0.11 m), with widths ranging from 300 to 400 m and lengths ranging from 450 to 840 m. The main lake characteristics are listed in Table 1 and Supplementary Table 1.

### DNA extraction

Total DNA extraction was carried out using lyophilized material (0.5 g) from each lake sample using the PowerLyzer PowerSoil DNA isolation kit (Qiagen, Venlo, Netherlands) according to the manufacturer’s protocol. The amount and quality of DNA were measured by 1% (w/v) agarose gel electrophoresis, and the final concentrations were quantified with a Qubit (Qubit® 2.0 Fluorometer, Life Technologies).

### Sequencing of DNA from water from soda lakes

A total of 24 samples were shotgun-sequenced (four lakes × three replicates × two seasons). Libraries were generated using the Nextera XT DNA Sample Preparation kit for paired-end fragments of 100 bp and sequenced on the Illumina HiSeq 2500 platform (Illumina, Inc., San Diego, CA, USA) following the manufacturer’s recommendations. Sequencing procedures were performed at the Laboratory for Functional Genomics Applied to Agriculture (http://www.esalq.usp.br/genomicafuncional/) located at the Luiz de Queiroz College of Agriculture (University of São Paulo, Piracicaba, SP, Brazil).

### Sequence analysis

The quality of the raw sequences was checked via FastQC 0.10.1 (http://www.bioinformatics.babraham.ac.uk/projects/fastqc/). The merging pair-end reads were realized using the pair-end read mergeR (PEAR) software^39^. After the merging process, sequences smaller than 50 bp and Phred < 20 were removed using Seqyclean 1.3.12 (http://cores.ibest.uidaho.edu/software/seqyclean)^40^. The resulting sequences were uploaded to the MG-RAST bioinformatics pipeline (http: //metagenomics. anl. gov) for analysis^41^.

### Data availability

These datasets were publicly available at MG-RAST (http://metagenomics.anl.gov). Sequences derived from the dry season are available under the codes [04SR_ET (mgm4810104.3, mgm4810097.3, and mgm4810100.3), 06SR_OT (mgm4810099.3, mgm4810102.3, and mgm4810101.3), 07SR_CVO (mgm4810094.3, mgm4810095.3 and, mgm4810107.3), 08SR_ET (mgm4810106.3, mgm4810093.3, and mgm4810096.3)] while the sequences derived from the wet season are available under the code mgp88859.

### The taxonomic and functional analysis

Taxonomic profiling of the microbiomes based on ribosomal RNA sequences was performed using the databases available in MG-RAST (Annotation source: Silva database (ssu); Max e-value cutoff: 1e−5, min % identity cutoff: 60%). To avoid errors associated with the difference in the sequencing depth of samples, they were normalized using the package edgeR (library size)^42^ in R software^43^.

Taxonomic patterns were compared among the lake microbiomes based on principal correspondence analysis (PCoA) considering the Bray–Curtis distance using the vegan package (vegdist, betadisper, and anosim)^44^. The differential abundance of taxa was evaluated using the edgeR package. The counts were normalized using the relative log expression (RLE) method, and the *p* values were corrected using the Benjamini–Hochberg (BH) method^45^. All graphics were generated using the ggplot2 package^46^.

Functional annotation was performed using the databases available in MG-RAST (Annotation source: SEED database; Max e-value cutoff: 1e−5, min % identity cutoff: 60%). Functional grouping was evaluated by heatmap construction using the function qplot on the ggplot2 package^46^. The enrichment of traits was calculated using differential expression analysis based on negative binomial distribution using the edgeR package^45^. This analysis was carried out using two datasets: (i) the whole bacterial community and (ii) the bacterial community, except for the phylum Cyanobacteria. Then, the enriched traits were grouped according to life history strategy (Y, A, or S strategy) as described by Malik et al.^4^. The classifications are presented in Supplementary Table 1.

### Correlation between abiotic and biotic features of the lakes

To understand the correlations between abiotic (physical and chemical features) and biotic (bacterial community composition) factors, we applied the db-RDA analysis followed by the forward selection test using the function ordistep in the vegan package. Thus, we determined the set of parameters that best explained the variation in community composition^47^. These selected parameters were correlated with the differentially abundant bacterial taxa using the package corrplot^48^.

### Heterotrophic prokaryotes biomass

The heterotrophic bacterial biomass (HBB) was estimated by heterotrophic prokaryote counts (cells mL^−1^) using flow cytometry (FCM) (BD Accuri C6). Samples of 1.2 mL of lake water were fixed with formaldehyde (1% final concentration), flash-frozen in liquid nitrogen, and stored at − 80 °C until analysis in the laboratory. Samples were stained with SYBR Green I at a final concentration of 1:10,000 for 15 min in the dark at room temperature. HP detection was evaluated using the FL1 channel (533 nm) of the fluorescence sets following the Gasol and del Giorgio^48^ protocol adapted for freshwater. The bacterial cell size (*V*) (in µm^3^ cell^−1^) was estimated using the relationship between the average bacterial cell size and the average fluorescence of the sample relative to the beads (FL1 bacteria/FL1 beads), as reported previously by Gasol and Del Giorgio (2000), as follows: V = 0.0075 + 0.11 × (FL1 bacteria/FL1 beads). The bacterial biomass (BB) (in pg C cell^−1^) was calculated by using the carbon-to-volume (V) (in µm^3^ cell^−1^) relationship derived previously by Norland^49^ from the data of Simon and Azam^50^, as follows: BB 0.12 × V^0.7^.

## Supplementary Information

Supplementary Information.
