# Supplementary material for: Disentangling the lifestyle of bacterial communities in tropical soda lakes
Source: Sci Rep. 2022 May 13;12:7939. doi: 10.1038/s41598-022-12046-2 (PMC9106740; doi:10.1038/s41598-022-12046-2)
Supplement: Supplementary file 1 — Supplementary Information. [file 41598_2022_12046_MOESM1_ESM.docx]

**Supplementary Material**

**Supplementary Table 01: Nutrient content of shallow alkaline lakes**

|  | 04SR_D | 06SR_D | 07SR_D | 08SR_D | 04SR_W | 06SR_W | 07SR_W | 08SR_W |
| --- | --- | --- | --- | --- | --- | --- | --- | --- |
| K  (mg.L-1) | 1,739 ± 99^Aa^ | 959.4 ± 161^Ab^ | 188.4 ± 15^Ac^ | 1,680 ± 75^Aa^ | 157.75 ± 45^Ba^ | 50.69 ± 13^Bb^ | 46.2 ± 5.99^Bb^ | 45.55 ± 10^Bb^ |
| Al  (mg.L-1) | 0.31 ± 0.04^Aab^ | 1.16 ± 0.81^Aa^ | 0.009 ± 0.002^Ab^ | 0.15 ± 0.01^Aab^ | 0.009 ± 0.003^Bb^ | 9.38 ± 0.16^Ba^ | 0.007 ± 0.004^Bb^ | 0.04 ± 0.007^Bb^ |
| B  (mg.L-1) | 6.03 ± 0.09^Ab^ | 2.02 ± 0.20^Ac^ | 0.29 ± 0.01^Ad^ | 10.87 ± 0.21^Aa^ | 0.51 ± 0.007^Ba^ | 0.09 ± 0.002^Bc^ | 0.094 ± 0.002^Bc^ | 0.29 ± 0.003^Bb^ |
| Cr  (mg.L-1) | 0.01 ± 0.0003^Aa^ | 0.01 ± 0.0009^Ab^ | 0.011 ± 0.0004^Ab^ | 0.013 ± 0.001^Ab^ | 0.001 ± 0.00001^B^ | 0.006 ± 0^B^ | 0.001 ± 0^B^ | 0.001 ± 0^B^ |
| Cu  (mg.L-1) | 0.04 ± 0.008^Ab^ | 0.088 ± 0.01^Aa^ | 0.01 ± 0.0008^Ac^ | 0.04 ± 0.004^Ab^ | 0.0001 ± 0^Bc^ | 0.002 ± 0^Ba^ | 0.009 ± 0^Bb^ | 0.0002 ± 0.0001^Bc^ |
| Fe  (mg.L-1) | 0.27 ± 0.03^Aab^ | 0.93 ± 0.58^Aa^ | 0.06 ± 0.001^Ab^ | 0.17 ± 0.01^Ab^ | 0.14 ± 0.01^Bb^ | 41.66 ± 0.72^Ba^ | 0.07 ± 0.009^Bb^ | 0.27 ± 0.021^Bb^ |
| Mg  (mg.L-1) | 138.2 ± 55^Bc^ | 192.7 ± 160^Bd^ | 43.52 ± 6^Ba^ | 203.1 ± 95^Bb^ | 23.65 ± 1.36^A^ | 18.95 ± 15^A^ | 20.22 ± 3.80^A^ | 9.47 ± 6.5^A^ |
| Mn  (mg.L-1) | 0.12 ± 0.009^Ab^ | 0.22 ± 0.08^Ab^ | 0.87 ± 0.07^Aa^ | 0.21 ± 0.003^Ab^ | 0.009 ± 0.0004^Bd^ | 1.60 ± 0.04^Ba^ | 0.07 ± 0.006^Bb^ | 0.03 ± 0.003^Bc^ |
| Ni  (mg.L-1) | 0.09 ± 0.001^Aa^ | 0.06 ± 0.008^Ac^ | 0.02 ± 0.001^Ad^ | 0.08 ± 0.002^Ab^ | 0.004 ± 0.0002^Bb^ | 0.02 ± 0.0003^Ba^ | 0.002 ± 0^Bd^ | 0.003 ± 0^Bc^ |
| Si  (mg.L-1) | 207.3 ± 10^Aa^ | 100.2 ± 7^Ac^ | 64.84 ± 4^Ad^ | 173.42 ± 6^Ab^ | 35.82 ± 0.64^Bb^ | 90.79 ± 2.59^Ba^ | 27.46 ± 0.21^Bd^ | 31.97 ± 0.54^Bc^ |
| Zn  (mg.L-1) | 0.07 ± 0.01^Aa^ | 0.03 ± 0.002^Ab^ | 0.015 ± 0.006^Ab^ | 0.06 ± 0.007^Aa^ | 0.004 ± 0.001^Bb^ | 0.068 ± 0.067^Ba^ | 0.013 ± 0.012^Bab^ | 0.01 ± 0.009^Bb^ |

The letter D correspond to the dry season while the letter W correspond to the wet season. The uppercase letters compare the seasons (dry and wet), while lowercase letters compare the lakes (P < 0.05).

**Supplementary table 02: The relative abundance of bacterial functions used in trait-based framework**

| Functions (level_2) | Strategies | 04SR_W | 06SR_W | 07SR_W | 08SR_W |
| --- | --- | --- | --- | --- | --- |
| ABC transporters | A | 0,96112% | 1,07783% | 0,89794% | 0,96231% |
| Chemotaxis, response regulators | A | 0,00756% | 0,00270% | 0,00275% | 0,00243% |
| Flagella protein? | A | 0,00763% | 0,00598% | 0,00372% | 0,00499% |
| Flagellar motility in Prokaryota | A | 0,43384% | 0,43459% | 0,47299% | 0,46463% |
| Glycoside hydrolases | A | 0,05197% | 0,01715% | 0,01844% | 0,03559% |
| Protein degradation | A | 1,49750% | 1,54603% | 1,48674% | 1,48713% |
| Protein secretion system, Type I | A | 0,02826% | 0,02027% | 0,01466% | 0,01815% |
| Protein secretion system, Type II | A | 0,25779% | 0,22238% | 0,14678% | 0,18302% |
| Protein secretion system, Type VI | A | 0,12443% | 0,10431% | 0,09385% | 0,10053% |
| Protein secretion system, Type VII (Chaperone/Usher pathway, CU) | A | 0,00272% | 0,00064% | 0,00022% | 0,00007% |
| Protein secretion system, Type VIII (Extracellular nucleation/precipitation pathway, ENP) | A | 0,02028% | 0,01963% | 0,01304% | 0,01479% |
| Protein translocation across cytoplasmic membrane | A | 0,56705% | 0,69843% | 0,80048% | 0,64979% |
| Selenoproteins | A | 0,20651% | 0,18314% | 0,21250% | 0,19839% |
| Siderophores | A | 0,02959% | 0,02234% | 0,01607% | 0,03144% |
| Sugar Phosphotransferase Systems, PTS | A | 0,02754% | 0,03709% | 0,07216% | 0,04032% |
| Social motility and nonflagellar swimming in bacteria | A | 0,00130% | 0,00170% | 0,00060% | 0,00039% |
| Sulfatases and sulfatase modifying factor 1 (and a hypothetical) | A | 0,06045% | 0,06443% | 0,02805% | 0,05416% |
| Two related proteases | A | 0,08744% | 0,09291% | 0,07671% | 0,07900% |
| Uni- Sym- and Antiporters | A | 0,27323% | 0,14784% | 0,04179% | 0,17868% |
| proteosome related | A | 0,12110% | 0,16224% | 0,09667% | 0,14615% |
| Alanine, serine, and glycine | Y | 0,77354% | 0,82245% | 0,76680% | 0,76311% |
| Aminosugars | Y | 0,12016% | 0,09153% | 0,08394% | 0,09632% |
| Arginine; urea cycle, polyamines | Y | 1,64515% | 1,62220% | 1,55899% | 1,64002% |
| Aromatic amino acids and derivatives | Y | 1,19506% | 1,35399% | 1,40759% | 1,30283% |
| Branched-chain amino acids | Y | 1,84284% | 2,18258% | 1,92727% | 1,90348% |
| CO2 fixation | Y | 0,99864% | 0,79580% | 0,81217% | 0,97911% |
| Carbohydrates | Y | 0,14117% | 0,08830% | 0,07230% | 0,10156% |
| Central carbohydrate metabolism | Y | 4,68262% | 4,66847% | 4,81967% | 4,56826% |
| Di- and oligosaccharides | Y | 0,79279% | 0,50201% | 0,41891% | 0,52569% |
| Fatty acids | Y | 0,99648% | 0,99746% | 0,87356% | 0,94028% |
| Fermentation | Y | 0,79497% | 0,73299% | 0,75746% | 0,78237% |
| Glutamine, glutamate, aspartate, asparagine; ammonia assimilation | Y | 0,88286% | 0,99474% | 1,00091% | 0,90916% |
| Lysine Biosynthesis | Y | 0,00512% | 0,01267% | 0,01600% | 0,00989% |
| Monosaccharides | Y | 0,88125% | 0,87562% | 0,93990% | 0,77586% |
| One-carbon Metabolism | Y | 1,22250% | 1,41392% | 1,18962% | 1,27644% |
| Organic acids | Y | 0,63920% | 0,72346% | 0,84469% | 0,64958% |
| Phospholipids | Y | 0,35651% | 0,33456% | 0,34538% | 0,34989% |
| Proline and 4-hydroxyproline | Y | 0,15546% | 0,13162% | 0,13526% | 0,13668% |
| Protein biosynthesis | Y | 6,56495% | 7,73691% | 7,98019% | 7,03897% |
| Purines | Y | 1,87679% | 2,07413% | 2,05920% | 1,96454% |
| Pyrimidines | Y | 1,02893% | 1,07342% | 1,07920% | 1,07258% |
| Sugar alcohols | Y | 0,37328% | 0,42103% | 0,34419% | 0,31201% |
| Capsular and extracellular polysacchrides | S | 1,45248% | 1,25259% | 1,37154% | 1,35963% |
| Cell wall of Mycobacteria | S | 0,13308% | 0,12587% | 0,11714% | 0,12261% |
| DNA recombination | S | 0,09583% | 0,11606% | 0,13084% | 0,11293% |
| DNA repair | S | 2,55365% | 2,66030% | 2,60237% | 2,55469% |
| Gram-Negative cell wall components | S | 1,03300% | 1,16187% | 1,41878% | 1,32808% |
| Gram-Positive cell wall components | S | 0,07845% | 0,06231% | 0,05418% | 0,06635% |
| Osmotic stress | S | 0,14110% | 0,16784% | 0,12469% | 0,15239% |
| Oxidative stress | S | 0,93982% | 0,90574% | 0,77174% | 0,94541% |
| Periplasmic Stress | S | 0,04570% | 0,05827% | 0,05924% | 0,05156% |
| Spore DNA protection | S | 0,00002% | 0,00015% | 0,00008% | 0,00007% |

*Continuation of Supplementary Table 02*

| Functions (level_2) | Strategies | 04SR_D | 06SR_D | 07SR_D | 08SR_D |
| --- | --- | --- | --- | --- | --- |
| ABC transporters | A | 0,85403% | 0,98740% | 1,13964% | 1,05031% |
| Chemotaxis, response regulators | A | 0,01610% | 0,00543% | 0,00400% | 0,01680% |
| Flagella protein? | A | 0,01134% | 0,00834% | 0,00352% | 0,01024% |
| Flagellar motility in Prokaryota | A | 0,48978% | 0,51010% | 0,42647% | 0,86871% |
| Glycoside hydrolases | A | 0,05557% | 0,02838% | 0,01709% | 0,04120% |
| Protein degradation | A | 1,51058% | 1,60484% | 1,49557% | 1,39779% |
| Protein secretion system, Type I | A | 0,01654% | 0,01855% | 0,01571% | 0,02826% |
| Protein secretion system, Type II | A | 0,21799% | 0,32864% | 0,15559% | 0,18557% |
| Protein secretion system, Type VI | A | 0,09505% | 0,13435% | 0,09674% | 0,07521% |
| Protein secretion system, Type VII (Chaperone/Usher pathway, CU) | A | 0,00052% | 0,00033% | 0,00047% | 0,00142% |
| Protein secretion system, Type VIII (Extracellular nucleation/precipitation pathway, ENP) | A | 0,03129% | 0,02278% | 0,02262% | 0,01901% |
| Protein translocation across cytoplasmic membrane | A | 0,57434% | 0,63160% | 0,75811% | 0,55188% |
| Selenoproteins | A | 0,23813% | 0,21945% | 0,20882% | 0,21127% |
| Siderophores | A | 0,02815% | 0,02437% | 0,02690% | 0,02100% |
| Sugar Phosphotransferase Systems, PTS | A | 0,03556% | 0,03443% | 0,07076% | 0,04847% |
| Social motility and nonflagellar swimming in bacteria | A | 0,00116% | 0,00094% | 0,00061% | 0,00122% |
| Sulfatases and sulfatase modifying factor 1 (and a hypothetical) | A | 0,03110% | 0,05767% | 0,03077% | 0,01336% |
| Two related proteases | A | 0,08909% | 0,09285% | 0,12534% | 0,12579% |
| Uni- Sym- and Antiporters | A | 0,32184% | 0,27457% | 0,09686% | 0,34468% |
| proteosome related | A | 0,07984% | 0,16878% | 0,07633% | 0,06475% |
| Alanine, serine, and glycine | Y | 0,78665% | 0,81430% | 0,78760% | 0,69098% |
| Aminosugars | Y | 0,16188% | 0,09347% | 0,08069% | 0,17617% |
| Arginine; urea cycle, polyamines | Y | 1,65692% | 1,60326% | 1,53643% | 1,96168% |
| Aromatic amino acids and derivatives | Y | 1,20983% | 1,30463% | 1,29586% | 1,22505% |
| Branched-chain amino acids | Y | 1,77924% | 2,00840% | 1,56767% | 1,30903% |
| CO2 fixation | Y | 0,93271% | 0,91472% | 0,83122% | 0,99754% |
| Carbohydrates | Y | 0,15265% | 0,09882% | 0,10194% | 0,15965% |
| Central carbohydrate metabolism | Y | 4,39897% | 4,74127% | 4,68432% | 4,01332% |
| Di- and oligosaccharides | Y | 0,97609% | 0,76662% | 0,69008% | 0,91631% |
| Fatty acids | Y | 1,00877% | 1,02947% | 0,92117% | 0,87048% |
| Fermentation | Y | 0,80113% | 0,77138% | 0,71230% | 0,72539% |
| Glutamine, glutamate, aspartate, asparagine; ammonia assimilation | Y | 0,88620% | 0,86412% | 0,89040% | 0,78785% |
| Lysine Biosynthesis | Y | 0,00614% | 0,00771% | 0,02186% | 0,00828% |
| Monosaccharides | Y | 0,73403% | 0,82917% | 0,77543% | 0,77543% |
| One-carbon Metabolism | Y | 1,14219% | 1,26617% | 1,03815% | 0,85448% |
| Organic acids | Y | 0,61931% | 0,64390% | 0,59017% | 0,56268% |
| Phospholipids | Y | 0,33825% | 0,38604% | 0,38177% | 0,35039% |
| Proline and 4-hydroxyproline | Y | 0,13044% | 0,20999% | 0,09096% | 0,11483% |
| Protein biosynthesis | Y | 6,62489% | 7,02775% | 8,34375% | 6,88505% |
| Purines | Y | 1,82383% | 1,97983% | 1,98987% | 1,71585% |
| Pyrimidines | Y | 1,01186% | 1,02363% | 1,09710% | 1,00425% |
| Sugar alcohols | Y | 0,35666% | 0,60021% | 0,33133% | 0,52924% |
| Capsular and extracellular polysacchrides | S | 1,52941% | 1,21912% | 1,35995% | 1,20095% |
| Cell wall of Mycobacteria | S | 0,12200% | 0,13321% | 0,12123% | 0,09034% |
| DNA recombination | S | 0,10114% | 0,10166% | 0,12912% | 0,10941% |
| DNA repair | S | 2,65976% | 2,65847% | 2,82956% | 2,67760% |
| Gram-Negative cell wall components | S | 0,98011% | 0,98003% | 1,36749% | 0,98178% |
| Gram-Positive cell wall components | S | 0,08524% | 0,08127% | 0,05908% | 0,08005% |
| Osmotic stress | S | 0,19193% | 0,18386% | 0,10109% | 0,21633% |
| Oxidative stress | S | 0,95621% | 0,92672% | 0,79168% | 0,91717% |
| Periplasmic Stress | S | 0,04523% | 0,05915% | 0,05756% | 0,05301% |
| Spore DNA protection | S | 0,00067% | 0,00038% | 0,00128% | 0,00219% |

All the significant functions used in the trait-based framework were represented here.

**Supplemetary Figure 1**

**Supplementary Figure 2**

**Supplementary Figure 3**

**Supplementary Figure 4**

**
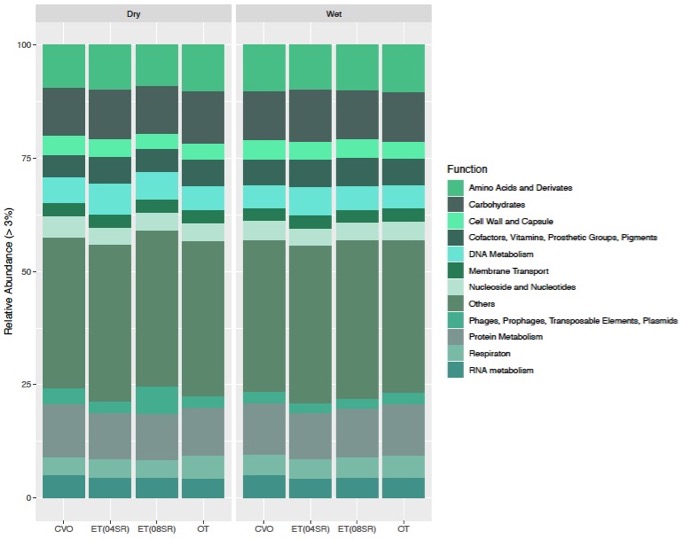
**

**Supplementary Figure 5**

**Legends**

**Supplementary Figure 01:** The relative abundance of the main bacterial phyla founded on shallow alkaline lakes.

**Supplementary Figure 02:** The differential abundance of bacterial groups during the dry season (p < 0.05).

**Supplementary Figure 03:** The differential abundance of bacterial groups during the wet season (p < 0.05).

**Supplementary Figure 04:** The relative abundance of the main bacterial functions founded on shallow alkaline lakes.

**Supplementary Figure 05:** The bacterial community traits-based analysis excluding the Cyanobacteria phylum. The heatmaps (letters A and C) represent the number of traits affiliated with each life strategy, while the boxplot represents the biomass of heterotrophic bacteria (letters B and D). The letters A and B represent the dry season, while the letters C and D represent the wet season.
